# The Stiffness of Cardiac Fibroblast Substrates Exerts a Regulatory Influence on Collagen Metabolism via α2β1 Integrin, FAK and Src Kinases

**DOI:** 10.3390/cells10123506

**Published:** 2021-12-11

**Authors:** Małgorzata Gałdyszyńska, Paulina Radwańska, Jacek Szymański, Jacek Drobnik

**Affiliations:** 1 Laboratory of Connective Tissue Metabolism, Department of Pathophysiology, Medical University of Lodz, Żeligowskiego 7/9, 90-752 Lodz, Poland; malgorzata.galdyszynska@umed.lodz.pl (M.G.); paulina.radwanska@umed.lodz.pl (P.R.); 2 Central Scientific Laboratory, Medical University of Lodz, Mazowiecka 6/8, 92-215 Lodz, Poland; jacek.szymanski@umed.lodz.pl

**Keywords:** collagen, cell culture, cardiac fibroblasts, focal adhesion kinase, heart, integrin α2β1, metalloproteinase, Src kinase

## Abstract

Information about mechanical strain in the extracellular space is conducted along collagen fibers connected with integrins and then transmitted within cells. An aim of the study is to verify the hypothesis that the stiffness of cardiac human fibroblast substrates exerts a regulatory effect on collagen metabolism via integrin α2β1 and downstream signaling. The experiments were performed on human cardiac fibroblasts cultured on stiff or soft polyacrylamide gels. Extracellular and intracellular collagen content, metalloproteinase-1 (MMP-1), metalloproteinase-9 (MMP-9) and expression of the α1 chain of the procollagen type I gene (*Col1A1*) were elevated in cultures settled on soft substrate. The substrate stiffness did not modify tissue inhibitors of matrix metalloproteinase capacity (TIMPs 1–4). Integrin α2β1 inhibition (TC-I 15) or α2 subunit silencing resulted in augmentation of collagen content within the culture. Expression of *Col1A1* and *Col3A1* genes was increased in TC-I 15-treated fibroblasts. Total and phosphorylated levels of both FAK and Src kinases were elevated in fibroblasts cultured on stiff substrate. Inhibition of FAK (FAK kinase inhibitor 14) or Src kinase (AZM 47527) increased collagen content within the culture. The substrate stiffness exerted a regulatory influence on collagen metabolism via integrin α2β1 and its downstream signaling (FAK and Src kinases) in cardiac fibroblasts.

## 1. Introduction

The heart comprises several types of cells, including cardiomyocytes, cardiac fibroblasts and smooth muscle cells [1,2]. To function correctly as a pump, the cardiomyocytes provide contractility; these cells are knitted together by connective tissue scaffolding [1,3]. Cardiac fibroblasts account for 70% of heart cells; they are involved in the regulation of extracellular matrix metabolism (ECM) and release cytokines that may induce cardiomyocyte hypertrophy [4]. Fibroblasts are also responsible for the transmission of electrical impulses between cardiomyocytes [1,2]. Cardiac fibroblasts also detect physical and chemical stimuli, and this may determine their function [3,5,6]

Several myocardial diseases are characterized by fibrosis, an excessive accumulation of collagen that leads to changes in the composition and structure of heart connective tissue. Fibrotic lesions are usually associated with repair processes and are accompanied by inflammation [3,7]. Two main types of heart fibrosis exist: reactive fibrosis, caused by the deposition of collagen in the interstitial space, and replacement fibrosis, which develops after damage to cardiomyocytes [8]. Excessive extracellular matrix accumulation modifies the mechanical properties of the heart, leading to an abnormal distribution of mechanical forces [3]. The synthesis of collagen is accompanied by secretion of matrix metalloproteinases (MMPs) [1,2].

Information about mechanical strain in the extracellular space is conducted along collagen fibers connected with integrins, and then transmitted within cells via downstream signaling [9,10,11]. Integrin receptors consist of a heterodimer receptor, comprising one α- and one β-subunit, that can bind a wide spectrum of ligands [12,13]. The composition of the receptors varies between tissue types [12]. Some collagen-binding integrins (α1β1, α2β1 and α11β1) are expressed on cardiac fibroblasts [5,12,14]. Moreover, changes in substrate stiffness have been found to influence the secretion of interleukin-6 (IL-6) by cardiac fibroblasts via α2β1 integrin and Src kinase [5]. It is believed that IL-6 is involved in the regulation of fibrosis [15,16,17,18].

The mechanical properties of the extracellular environment are dynamic and they are continuously sensed by resident cells [19]. The stiffness of the extracellular environment influences many cell functions, including cytokine release [5], proliferation [20], transdifferentiation to myofibroblasts [21] and migration [19]. The aim of the present study is to determine whether the stiffness of the cell substrate influences the regulation of collagen metabolism by cardiac fibroblasts. This paper evaluates the effects of mechanical stimuli on both the synthesis and degradation of collagen. It also examines the involvement of α2β1 integrin in the transmission of the signal participating in collagen metabolism regulation and determines whether both FAK and Src kinases are required for regulation collagen metabolism.

## 2. Materials and Methods

### 2.1. Polyacrylamide Gel Preparation

To mimic the different mechanical properties of the cellular environment, polyacrylamide gels with varying hardness were prepared (stiff and soft gel). The desired gels were produced using 40% polyacrylamide (final concentration 8%) (BioRad, Hercules, CA, USA) and 10 mm HEPES pH 8.5 (Sigma Aldrich, St. Louis, MO, USA); the stiff gels were prepared using 0.1% N,N’-methylene-bis-acrylamide, while the soft gels used 0.06% N,N’-methylene-bis-acrylamide (BioRad, Hercules, CA, USA). The solutions were polymerized for about 30 min between sterile glass plates using ammonium persulfate (10%; Sigma Aldrich, St. Louis, MO, USA) and N,N,N’,N’-tetramethylenediamine (Sigma Aldrich, St. Louis, MO, USA). After polymerization, the plates were separated, and 2.5 cm diameter gel disks were cut and placed into 6-well culture plates for further experiments.

The gels were activated by the addition of 0.5 mm sulfo-SANPAH (Sigma Aldrich, St. Louis, MO, USA) in solution containing 50 mm HEPES pH 8.5 and 0.5% DMSO on the surface of each gel disk, and then exposed to UV light for 30 min. The activated gels were washed for three times with 50 mm HEPES pH 8.5 (Sigma Aldrich, St. Louis, MO, USA) to eliminate any remaining sulfo-SANPAH. Following this, the gels were coated with a 100 µg/mL solution of type I collagen (Sigma Aldrich, St. Louis, MO, USA) in 0.1 m acetic acid and incubated overnight at 4 °C. Excess acetic acid was removed before use by washing with HBSS (Thermo Fisher Scientific, Waltham, MA, USA). The mean elastic modulus values were equal to 2.23 ± 0.8 kPa for the soft gel and 8.28 ± 1.06 kPa for the hard gel, as described previously [5]. The soft gel demonstrated a similar rigidity to cardiac fibroblasts isolated from the ventricle [22], whereas the stiff gel was significantly more rigid. Both gels used in the present paper, i.e., soft (2.23 + 0.8 kPa) and stiff (8.28 + 1.06 kPa) reflected the physiological range of the elastic modulus of healthy human heart tissue [22].

### 2.2. Cell Culture

This research was performed on an immortalized human cardiac fibroblast cell line (cat.no. T0446) obtained from ABM (Richmond, BC, Canada). According to the manufacturer’s information, the cells were immortalized by serial passaging and transduction with recombinant lentiviruses carrying the SV40 Large T antigen. The cells were cultured according to the manufacturer’s instructions in PriGrow IV medium (ABM, Richmond, BC, Canada) supplemented with 10% fetal bovine serum (FBS) (ABM, Richmond, BC, Canada), l-glutamine (ABM, Richmond, BC, Canada), penicillin/streptomycin solution (ABM, Richmond, BC, Canada), non-essential amino acids (NEAA; Sigma Aldrich, St. Louis, MO, USA), 5 µg/mL insulin (Thermo Fisher Scientific, Waltham, MA, USA) and 50 µg/mL vitamin C (Sigma Aldrich, St. Louis, MO, USA) on plates coated with 10 µg/cm^2^ type I collagen (Sigma Aldrich, St. Louis, MO, USA) at 37 °C, 5% CO_2_. Each experiment lasted for 96 h. The cell culture medium was collected into sterile Eppendorf tubes and centrifuged (1000× *g*, 10 min). The number of both total and necrotic cells (stained with trypan blue) was counted in a Bürker chamber. The analysis of the necrotic cell number did not confirm cytotoxic effects of gels or inhibitors.

### 2.3. Reagent Preparation

The following inhibitors were used: TC-I 15 (potent α2β1 integrin inhibitor), AZM 475,271 (Src kinase inhibitor) and FAK inhibitor 14 (FAK kinase inhibitor; Tocris, Ellisville, MO, USA). TC-I 15 and AZM 475,271 were dissolved in DMSO (Sigma Aldrich, St. Louis, MO, USA), diluted in medium and used at concentrations of 10^−7^ mol/L and 10^−8^ mol/L. Two control groups were used: the first was untreated controls (CTRs) and the second consisted of cells treated with 0.001% DMSO. FAK inhibitor 14 was dissolved in water, diluted in medium and used at concentrations of 10^−6^ mol/L and 10^−7^ mol/L, and then compared with untreated controls (CTRs). The medium was changed every day.

### 2.4. ITGA2 Gene Silencing

ITGA2 gene silencing was performed by means of siRNA (Dharmacon™, Lafayette, CO, USA). siRNA was transfected into cells using RNAiMAX reagent (Thermo Fisher Scientific, Waltham, MA, USA) according to the manufacturer’s instructions. Three groups were compared in this experiment: an intact control (CTR), cells treated with non-targeting siRNA (final concentration, 25 µM; NT) and cell cultures treated with siRNA to silence the ITGA2 gene (final concentration, 25 µM; ITGA2). Cells were incubated with siRNA for six hours; after this time, the culture medium was changed to full culture medium. The effectiveness of α2 integrin subunit silencing was confirmed by flow cytometry [5].

### 2.5. qPCR

Total RNA was isolated using a Total RNA Mini Kit minicolumn (A & A Biotechnology, Gdynia, Poland). RNA concentration was measured using a NanoDrop™ One Spectrophotometer (Thermo Fisher Scientific, Waltham, MA, USA). Following this, equal amounts of RNA (500 ng) from each group were transcribed into cDNA using PrimeScript RT-PCR Kit (Takara, Kusatsu, Shiga, Japan).

The amplification reactions were performed using the Universal Probe Library (UPL) (Roche, Indianapolis, IN, USA) and RealTime Ready Custom Single Assay (Roche, Indianapolis, IN, USA) based on Taqman probes (for *Col1A1*, *Col3A1*, *GAPDH*, *RPLP0*, *Ywhaz*). The reactions were conducted using FastStart Essential Probe Master (Roche, Indianapolis, IN, USA) according to the manufacturer’s protocol. The reactions were carried out using the following program: 95 °C for 10 min followed by 55 cycles of 95 °C for 10 s, 60 °C for 30 s and 72 °C for 1 s, followed by 40 °C for 30 s. Ribosomal protein 0 (*RPLP0*), tyrosine 3-monooxygenase/tryptophan 5-monooxygenase activation protein zeta (*Ywhaz*) and glyceraldehyde-3-phosphate dehydrogenase (*GAPDH*) were used as reference genes. Each reaction was conducted in duplicate and expressed as relative expression, calculated using LightCycler^®^ 96 software (Roche, Indianapolis, IN, USA).

### 2.6. Collagen Content

Both the extracellular and intercellular collagen content were estimated according to Wöessner’s method. Cell cultures and media were dried in a laboratory oven. The total collagen level was evaluated based on the hydroxyproline level by hydrolysis of dried samples with 6N HCl at 100 ℃ for 24 h. Hydrolysates were neutralized by 5N NaOH. Samples of 0.2 mL were taken for further analysis and diluted with redistilled water to a final volume of 1 mL. Hydroxyproline was oxidized to pyrrole by 0.5 mL of chloramine T in a citrate buffer (pH 6.0), then shaken and incubated for 20 min at room temperature. In order to remove excess chloramine T, 0.5 mL of 3.15 m perchloric acid was added. After 5 min incubation at room temperature, the samples were treated with 0.5 mL of 20% p-dimethylaminobenzaldehyde (Sigma Aldrich, St. Louis, MO, USA) and incubated in 60 ℃ water bath for 20 min. The optical density was measured at 560 nm on a spectrophotometer

### 2.7. Enzyme-Linked Immunosorbent Assay (ELISA)

Collagen metabolism parameters (MMP-1, MMP-2, MMP-9, TIMP 1-4) were measured using a specific ELISA kit (E-EL-H1441, E-EL-H1445, E-EL-H0184, E-EL-H1453, E-EL-H1454 and E-EL-H1455 Elabscience, Wuhan, China and RAB0372, Sigma Aldrich, St. Louis, MO, USA) in samples of cell culture media The content of Src and FAK kinase (total and phosphorylated) was analyzed using PEL-SRC-Y419-T and PEL-FAK-Y397-T kits (RayBiotech Life, Peachtree Corners, GA, USA) according to the manufacturer’s instructions. Before each assay, the samples were appropriately diluted. Absorbance was determined using an Epoch™ Microplate Spectrophotometer (BioTek Instruments Inc., Winooski, VT, USA).

### 2.8. Statistical Analysis

Results were presented as mean ±SD. The data were tested for normality using the Shapiro–Wilk test. Depending on its distribution, the data were tested using parametric (independent two-sample *t*-test or one-way ANOVA with Bonferroni post-test correction) or non-parametric tests (Mann–Whitney U-test or Kruskal–Wallis test). All analyses were performed using Statistica 13.1 software (StatSoft, Tulsa, OK, USA). Results with a *p*-value below 0.05 were regarded as statistically significant. All experiments were performed in at least three independent replicates.

## 3. Results

### 3.1. Evaluation of α2 Integrin Subunit Density in Cardiac Fibroblasts Cultured on Gels with Different Rigidities

**Figure 1:**
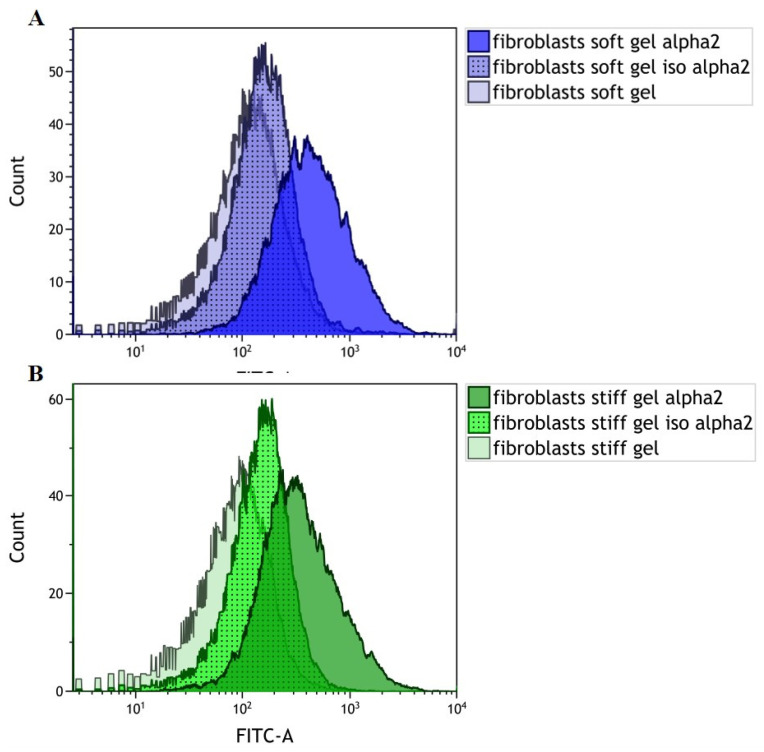
FACS profile showing the expression of the α2 integrin subunit in cardiac fibroblasts cultured on soft (**A**) and stiff (**B**) substrate (right curve) compared to isotype controls (middle curve) and cellular autofluorescence controls (left curve).

Our findings indicate that α2 integrin subunit expression occurs in cardiac fibroblasts settled on both soft and stiff gels (Figure 1A,B). The obtained FACS profile confirms the expression of the α2 integrin subunit within cardiac fibroblasts cultured on soft (Figure 1A) and stiff (Figure 1B) gels compared to isotype and cellular autofluorescence controls.

### 3.2. The Mechanical Properties of the Cell Environment Modulate Collagen Content in Cardiac Fibroblasts

**Figure 2:**
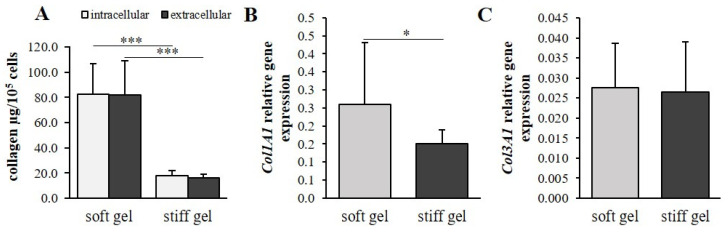
Intracellular and extracellular collagen content in cardiac fibroblasts and cardiac fibroblast media cultured on soft and stiff gels (**A**). Relative expression of *Col1A1* (**B**) and *Col3A1* (**C**) genes in cardiac fibroblast cultures on soft and stiff substrate. Each value represents mean ±SD (* *p* < 0.05; *** *p* < 0.001).

Human immortalized cardiac fibroblasts were cultured for 96 h on polyacrylamide gels of varying stiffness to mimic the different mechanical properties of the cellular environment. Both extracellular and intracellular collagen content were markedly elevated in cells cultured on soft substrate (*p* < 0.001; Figure 2A) compared with those on the stiff substrate. Interestingly, the α1 chain of the procollagen type I gene (*Col1A1*) demonstrated increased expression in cells cultured on soft gel (*p* < 0.05; Figure 2B); however, the α1 chain of the procollagen type III gene (*Col3A1*) demonstrated similar expression in the two substrates (Figure 2C).

**Figure 3:**
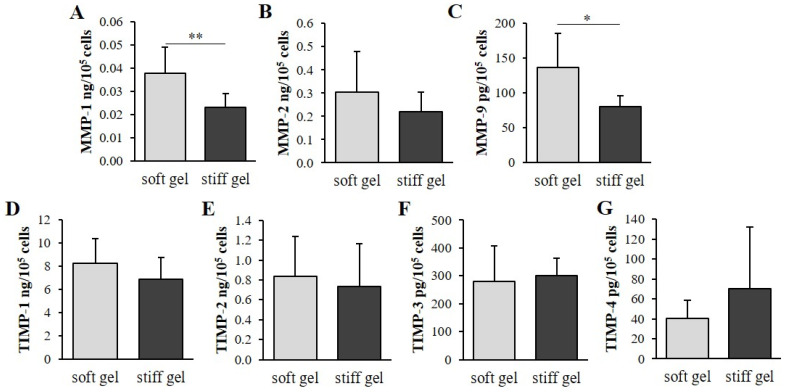
Content of MMP-1 (**A**), MMP-2 (**B**), MMP-9 (**C**), TIMP-1 (**D**), TIMP-2 (**E**), TIMP-3 (**F**) and TIMP-4 (**G**) within cardiac fibroblast media cultured on soft and stiff gels. Each value represents mean ±SD (* *p* < 0.05; ** *p* < 0.01).

Metalloproteinases (MMPs) and tissue inhibitors of metalloproteinases (TIMPs) were evaluated in culture media. The levels of MMP-1 and MMP-9 were found to be elevated in the soft cultures compared to the stiff cultures (*p* < 0.01; Figure 3A,C); however, no such difference was observed for MMP-2 (Figure 3B). In addition, no significant changes in TIMP-1, TIMP-2, TIMP-3 or TIMP-4 were observed between the soft and stiff gel cultures (Figure 3D–G).

### 3.3. The Effect of α2 Integrin Subunit Inhibition on Collagen Content in Vitro

**Figure 4:**
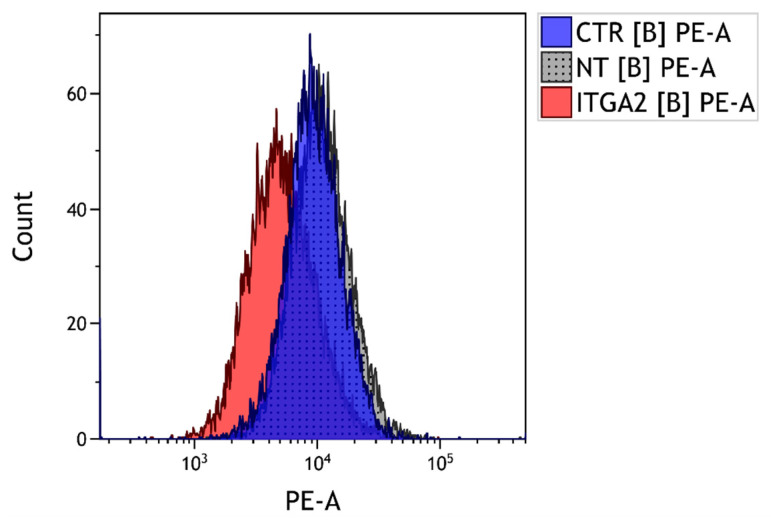
FACS profile showing the expression of the α2 integrin subunit in cardiac fibroblasts after silencing of the α2 integrin subunit (ITGA2; left curve), in cells treated with non-targeting siRNA (NT2; right curve) and in untreated controls (CTR; middle curve).

**Figure 5:**
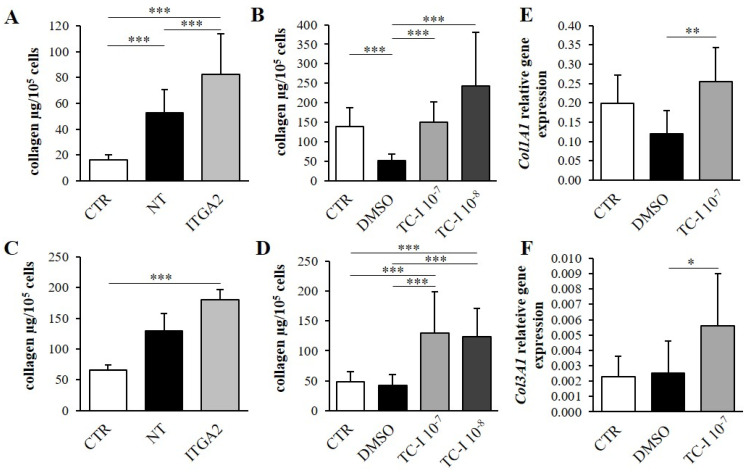
Total collagen content in cardiac fibroblasts cultured on soft (**A**) and stiff (**C**) gels after silencing of the α2 integrin subunit (ITGA2) in cells treated with non-targeting siRNA (NT) and in untreated controls (CTR). Effects of TCI 15 (α2β1 integrin inhibitor) applied at concentrations of 10^−7^ mol/L(TC-I 10^−7^) and 10^−8^ mol/L (TC-I 10^−8^), DMSO (TC-I 15 solvent) and untreated controls (CTR) on collagen content in cardiac fibroblasts cultured on soft (**B**) and stiff (**D**) gels. Relative expression of *Col1A1* (**E**) and *Col3A1* (**F**) genes in cardiac fibroblasts cultured on soft gel after administration of TC-I 15 at concentrations of 10^−7^ mol/L (TC-I 10^−7^), DMSO and untreated controls (CTR). Each value represents a mean ±SD (* *p* < 0.05; ** *p* < 0.01; *** *p* < 0.001).

The physical properties of the cellular environment are known to change the density of the α2 integrin subunit. The change in the level of the α2 integrin subunit was monitored by fluorescence-activated cell sorting (FACS; Figure 4). Previous studies have found that cardiac fibroblasts that settle on soft substrate demonstrate greater α2 integrin subunit density than those on a stiff gel [5]. The present study examined whether this integrin also influences collagen content in cardiac fibroblasts. A five-fold increase of collagen content was observed in α2 integrin-silenced fibroblasts cultured on soft substrate. A statistically-significant elevation (Figure 4A) of collagen capacity was observed in the α2 integrin-silenced cells compared with non-targeting siRNA fibroblasts (NT, *p* < 0.05) and untreated controls (CTR, *p* < 0.001). Treatment with non-targeting siRNA resulted in greater collagen accumulation in culture compared with untreated controls (*p* < 0.001; Figure 4A). In cells cultured on stiff gel, the collagen content in the α2 integrin-silenced cells was found to be 2.7-fold higher than in non-treated controls (*p* < 0.001; Figure 5C). No significant differences were observed between α2 integrin-silenced cells and non-targeting siRNA controls (Figure 5C).

The administration of TC-I 15 (a selective α2β1 integrin inhibitor) at concentrations of 10^−7^ mol/L and 10^−8^ mol/L was sufficient to increase the collagen content in cells cultured on soft gel compared with cultures treated with DMSO (TC-I 15 solvent; *p* < 0.001; Figure 5B). In the soft substrate cultures, DMSO administration was found to cause a decrease in collagen content compared with the non-treated group (*p* < 0.001; Figure 5B). Cultures settled on stiff substrate also demonstrated increased collagen content under the influence of 10^−7^ mol/L and 10^−8^ mol/L TC-I 15 in comparison with the DMSO-treated group (*p* < 0.001) and untreated controls (*p* < 0.001; Figure 5D). In both soft and stiff gel cultures, a similar accumulation of collagen was observed in both the control (CTR) and DMSO groups.

In cells cultured on soft gel, application of 10^−7^ mol/L TC-I 15 inhibitor increased the expression of the α1 chain of the procollagen type I (*Col1A1*) and III (*Col3A1*) genes as compared with the DMSO-treated group (*p* < 0.01, *p* < 0.05, respectively; Figure 5E,F).

### 3.4. Determination of the Effect of Mechanical Properties on α2β1 Integrin Second Messengers

**Figure 6:**
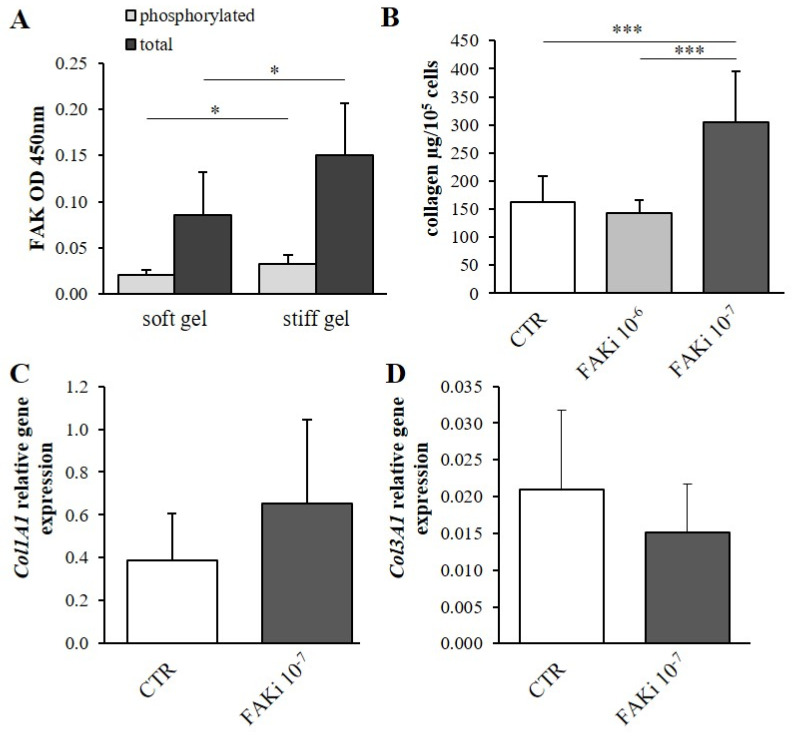
Difference in the total and phosphorylated focal adhesion kinase (FAK) levels between cardiac cells cultured on soft and stiff gels (**A**). Total collagen content in cardiac fibroblasts after administration of FAK inhibitor 14 (FAK kinase inhibitor) at concentrations of 10^−6^ mol/L(FAKi 10^−6^) and 10^−7^ mol/L (FAKi 10^−7^) compared with untreated controls (CTR) (**B**). Relative expression of *Col1A1* (**C**) and *Col3A1* (**D**) genes in cardiac fibroblasts after administration of FAK inhibitor 14 at a concentration of 10^−7^ mol/L (FAKi 10^−7^) compared to untreated controls (CTR). Each value represents a mean ±SD (* *p* < 0.05; *** *p* < 0.001).

**Figure 7:**
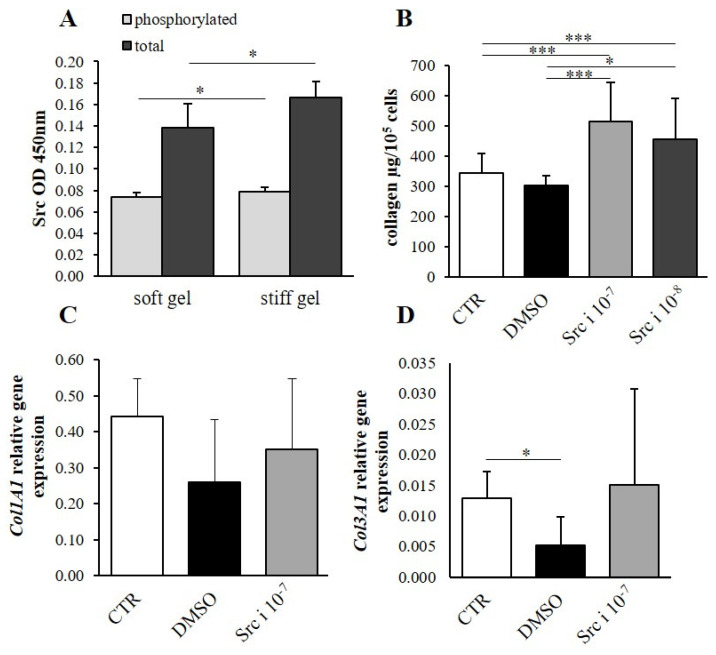
Differences in the levels of total and phosphorylated Src kinase (Src) in cardiac cells cultured on soft and stiff gels (**A**). Total collagen content in cardiac fibroblasts after administration of AZM 475,271 (Src kinase inhibitor) at concentrations of 10^−7^ mol/L (Src i 10^−7^) and 10^−8^ mol/L (Src i 10^−8^), DMSO (AZM 475,271 solvent) and untreated controls (CTR) (**B**). Relative expression of *Col1A1* (**C**) and *Col3A1* (**D**) genes in cardiac fibroblasts after administration of AZM 475,271 at a concentration of 10^−7^ mol/L (Src i 10^−7^) compared to DMSO and untreated controls (CTR). Each value represents a mean ± SD (* *p* < 0.05; *** *p* < 0.001).

The total phosphorylated and non-phosphorylated focal-adhesion kinase (FAK) and Src kinase content was evaluated. It was found that both the total and phosphorylated (active) forms of FAK were elevated in the stiff substrate cultures compared with the soft gel cultures (*p* < 0.05, Figure 6A); however, the levels of both the total and phosphorylated forms of Src kinase were elevated in the stiff gel cultures (*p* < 0.05, Figure 7A).

### 3.5. The Effect of Second Messenger Inhibition on Collagen Content

The previous section confirmed that information about substrate stiffness is transmitted by FAK and Src kinases (Figure 5A or Figure 6A). Hence, the next question was whether both FAK and Src kinase activity may determine collagen deposition within the fibroblast culture. The cells treated with FAK inhibitor were cultured on stiff substrate. It was found that treatment with FAK kinase inhibitor 14 did not influence collagen content in the culture compared with controls (CTR) when administered at 10^−6^ mol/L (FAKi 10^−6^); however, administration at 10^−7^ mol/L (FAKi 10^−7^) elevated collagen content as compared with non-treated controls (CTR, *p* < 0.001) and with the 10^−6^ mol/L FAK inhibitor variant (FAKi 10^−6^, *p* < 0.001).

Cells treated with FAK kinase inhibitor 14 at a concentration of 10^−7^ mol/L demonstrated a 1.9-fold increase in collagen content in comparison with untreated controls (*p* < 0.001; Figure 6B); however, no change was observed in the α1 chains of procollagen type I (*Col1A1*) and III (*Col3A1*) gene expression (Figure 6C,D).

AZM 475,271 (Src kinase inhibitor) administration at a concentration of 10^−8^ mol/L (Src i 10^−8^) increased collagen content as compared with controls (CTR, *p* < 0.001), as well as with the DMSO (inhibitor solvent)-treated group (*p* < 0.05, Figure 7B). Moreover, AZM 47,527 applied at a concentration of 10^−7^ mol/L (Src i 10^−7^) elevated collagen content compared with controls (CTR, *p* < 0.001) and DMSO-treated fibroblasts (DMSO, *p* < 0.001). On the other hand, Src kinase inhibitor (10^−7^ mol/L) did not influence the expression of the genes associated with the α1 chains of procollagen type I (*Col1A1*) and III (*Col3A1*; Figure 7C,D) compared to controls (CTR) and DMSO-administered cultures (DMSO). However, the application of DMSO caused a decrease in the expression of the *Col3A1* gene in comparison with non-treated controls (*p* < 0.05; Figure 7D). Fibroblasts treated with Src were cultured on a soft substrate.

## 4. Discussion

Our findings indicate that the stiffness of the cardiac fibroblast substrate may exert a regulatory influence on collagen content in cultures. Hence, fibrosis development could be dependent on the physical properties of the extracellular environment. These observations could suggest that collagen deposition is increased within the soft substrate culture (2.23 ± 0.8 kPa) with an elasticity similar to cardiac fibroblasts isolated from the ventricle [23]; markedly lower collagen accumulation was observed with the stiff surface. This data suggests that the rigidity of the cellular environment can be sensed by cardiac fibroblasts.

These cells also modify the physical properties of their extracellular spaces by collagen deposition. Fibrosis increases the stiffness of the extracellular space [24] and the intensity of this process may modify the rigidity of the cell environment. Similarly, *Col1A1* gene expression was found to be elevated. Thus, the signal induced by physical stimuli from the extracellular space is conducted to the nucleus and increases the expression of the α1 chain procollagen type I gene. This process, comprising the activation of the early transcriptional step of collagen synthesis, participates in fibrosis development. Contradictory findings have been obtained by studies examining fibrotic gene expression in fibroblasts settled on substrates with various stiffness; however, these studies used different models and experimental subjects. In such cases, maximal expression of *Col1A1* was reported in rat fibroblasts cultured on a substrate with a stiffness of 12 kPa, while MMP-13 expression decreased in those cultured on substrates with a stiffness of 2 kPa to 50 kPa. The mRNA expression of connective tissue growth factor increased with cell substrate stiffness. In this case, the rat fibroblasts were derived from hearts subjected to sham or aortocaval fistula surgery [25]. In contrast, human lung fibroblasts cultured on stiff matrix (20 kPa) demonstrated elevated fibronectin 1 and *Col1A1* gene expression [26]. In addition, rat-derived pulmonary arterial adventitial fibroblasts demonstrated upregulated *Col1A1*, *Col3A1* and elastin expression at higher substrate stiffness values [27].

Heart fibrosis is not only influenced by the synthesis of collagen but also by its breakdown. The cleavage of collagen is mainly dependent on matrix metalloproteinases (MMP) and their inhibitors (TIMPs). Cardiac fibroblasts produce and release MMP [28]. Our experiments examined the concentration of matrix metalloproteinase-1 (MMP-1)—also known as interstitial collagenase—which is known to degrade collagen fibers, and two gelatinases—MMP-2 and MMP-9—which participate in collagen cleavage in several cardiovascular diseases including hypertension [29], heart ischemia [30] and cardiac hypertrophy during pressure overload [31]. MMP-1 and MMP-9 levels were higher in the soft substrate cultures than in the stiff gel cultures. Riikonen et al. reported elevated MMP1 levels in human osteosarcoma cell lines with overexpressed integrin α2β1 [32], suggesting the involvement of α2β1 integrin in the regulation of MMP1 release. In the present study, the levels of all the investigated TIMPs, i.e., 1–4, remained unchanged by the physical properties of the substrate.

The observed augmentation of collagen deposition by soft gel suggests that the physical properties of the cell environment regulate fibrosis. In cultures settled on soft substrate, collagen synthesis was greater than its cleavage. The concurrent elevation of MMP-1 and 9, as well as the increased synthesis of collagen, may support heart remodeling. This hypothesis should be further investigated.

Inhibition of the α2β1 integrin via pharmacological blockade by TC-I 15 and by siRNA silencing of the α2 integrin subunit indicates that the α2β1 integrin is responsible for the regulation of collagen deposition. The intensity of downstream signaling associated with integrins (FAK and Src kinases) is modified by elasticity of the cellular substrate. Experimental inhibition of both focal-adhesion kinase (FAK) and Src kinases shows that they participate in the regulation of collagen deposition in cardiac fibroblast cultures. However, FAK or Src inhibition did not appear to have any effect on *Col1A1* and *Col3A1* gene expression, suggesting that procollagen gene expression is regulated by different signal pathways. Rajshankar et al. reported that FAK inhibits collagen degradation and influences its reorganization. FAK-null fibroblast culture demonstrates increased levels of both collagen and membrane-type MMP [33]. Moreover, FAK is reported to induce myofibroblast differentiation and recruitment, playing an important role in lung fibrosis [34]. Fibroblasts derived from hypertrophic scar tissue demonstrate greater FAK and Src activation compared to intact skin. Deactivation of both FAK and Src improves the ECM deposition in the cells of hypertrophic scars [35]. The above data suggests that Src or FAK activity regulates collagen metabolism mainly by influencing protein catabolism and the formation of profibrotic myofibroblasts.

To control for the non-specific effects of siRNA delivery, and to provide a baseline, non-targeting RNA (NT RNA) was applied. Our data show that the delivery of NT RNA not associated with target gene silencing significantly increased collagen content in cultures settled on the soft gel; however, this effect was not statistically significant in fibroblast cultures from stiff gel. These observations suggest that the effects of siRNA delivery to cells are dependent on the stiffness of the fibroblast substrate (Figure 5A,C). The effects of dimethyl sulfoxide (DMSO), the solvent for TC-I 15 and AZM 475,271 of the Src-inhibitor were tested in the control group. The results of DMSO application were found to be dependent on substrate stiffness; DMSO significantly (Figure 5B) or insignificantly (Figure 7B) decreased the collagen content on the soft gel, but had no clear effects on the stiff gel (Figure 5D). The effects of DMSO on collagen gene expression or this protein content have been reported previously [36,37].

Our findings support the hypothesis that fibroblasts not only sense the stiffness of the environment but may also adjust the physical properties of the substrate by the secretion and accumulation of collagen. The profibrotic signal could be based around the release of IL-6 from cardiac fibroblasts, which exerts a profibrotic effect within the heart [38,39]. The release of IL-6 by cardiac fibroblasts is regulated by the elastic properties of the substrate. Our previous findings indicate higher levels of IL-6 in fibroblasts cultured on soft gel compared to those on stiff gel [5]. This observation correlates with our present findings indicating greater augmentation of collagen content in soft gel cultures. In addition, both collagen accumulation and IL-6 release are dependent on α2β1 integrin activity. This integrin also takes part in the regulation of the collagen network in the dermis [40]. IL-6 promotes the differentiation of cardiac fibroblasts into myofibroblasts, accelerates their proliferation [38,39] and activates fibrosis [41]. Co-administration of IL-6 with its soluble receptor increased collagen accumulation within cardiac fibroblast cultures; however, injections of IL-6 in rats elevated the cardiac collagen volume fraction and heart stiffness [4]. IL-6 has also been found to have a profibrotic effect in other organs; inhibition of IL-6 release attenuates kidney fibrosis and decreases accumulation of fibroblasts [38]. In addition to regulating IL-6 release, the stiffness of the cell substrate may exert multiple effects on fibroblasts and collagen content via a range of processes. Ceccato et al. reported that cardiac fibroblasts derived from rat left ventricles settled on soft substrate (6 kPa) released elevated levels of TNFα (Tumor Necrosis Factor α), interleukin-3, interleukin-4 and interleukin-6. These compounds may also participate in the regulation of fibrosis [42].

## 5. Conclusions

The stiffness of the extracellular environment is sensed by human cardiac fibroblasts. The activity of integrin-dependent signaling molecules (FAK, Src) was here found to vary between fibroblasts cultured on substrates with different stiffness. The stiffness of the cell substrate exerted regulatory effects on collagen accumulation in cardiac fibroblast cultures. The information influencing collagen metabolism was transmitted by α2β1 integrin and downstream signaling processes, comprising focal adhesion kinase (FAK) and Src kinase. The physical properties of the cellular substrate exerted a pleiotropic effect on collagen metabolism. Therefore, the stiffness of the environment modified collagen synthesis at the transcriptional level, and increased the levels of MMP-1 and MMP-9 in culture.

**Figure 8:**
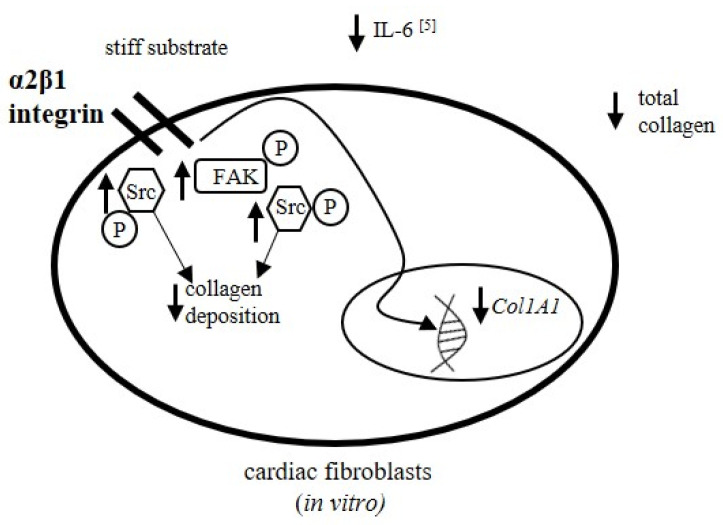
Model of the role of α2β1 integrin in the regulation of collagen deposition in cardiac fibroblast cultures. Lower collagen deposition was observed in cardiac fibroblasts settled on stiff substrate. Accumulation of total collagen and expression of the α1 chain of procollagen type I are dependent on α2β1 integrin. In addition, on the stiff gel, a higher integrin-dependent signal measured as phosphorylated FAK and Src kinases was observed. The release of profibrotic interleukin-6 (IL-6) from cardiac fibroblasts was decreased in the stiff gel cultures [5]. This effect is also dependent on α2β1 integrin.

These findings shows for the first time that the physical properties of the environment may play a regulatory role during heart fibrosis. In cases of heart hypertrophy or fibrosis, characterized by increased stiffness of the heart, changes in the physical properties of the heart muscle may control collagen deposition and determine its metabolism (Figure 8).

## Data Availability

The data that support the findings of this study are available from the corresponding author upon request.
